# Special Staining of the Liquid-Based Cytopathology Test in Bronchoalveolar Lavage Fluid for Diagnosis of Invasive Pulmonary Aspergillosis with Nonneutropenic Patients

**DOI:** 10.1155/2020/8243473

**Published:** 2020-03-31

**Authors:** Yue Hu, Lin Zheng, Deng Pan, Lei Shao, Xianfa Xu, Yiming Yu, Qidong Zhuang, Zaichun Deng, Zhongbo Chen

**Affiliations:** ^1^Department of Pulmonary and Critical Care Medicine, The Affiliated Hospital of Medical School of Ningbo University, 247 Renmin Road, Ningbo, Zhejiang 315020, China; ^2^Department of Microbiology, The Affiliated Hospital of Medical School of Ningbo University, 247 Renmin Road, Ningbo, Zhejiang 315020, China; ^3^Department of Cell, Clinicopathological Diagnosis Center of Ningbo, 685 Huancheng North Road, Ningbo, Zhejiang 315211, China

## Abstract

In recent years, various biomarkers have been gradually applied on bronchoalveolar lavage (BAL) fluid for the diagnosis of invasive pulmonary aspergillosis (IPA). The objective of this study is to assess the value of the liquid-based cytopathology test (LCT) for improving the identification of IPA in BAL fluid from possible IPA patients, following special staining with periodic acid-Schiff staining (PAS) or Grocott's methenamine silver (GMS). A total of 47 consecutive possible IPA patients who underwent bronchoscopy with BAL fluid from January 2017 to December 2018 were included. 45 people had a pair of BAL fluid specimens and 2 patients had two BAL fluid specimens. The 49 pairs of BAL fluid specimens were processed for culture, tuberculosis acid fast staining smear, direct microbial smear, and LCT with special staining (PAS and GMS), respectively. Then, we compared the sensitivity and specificity of PAS and GMS in BAL fluid in high-risk patients. Among 47 possible IPA patients, 25 patients had proven/probable IPA, and 11 patients had other invasive fungal diseases. The sensitivity of GMS was higher than that of PAS (92.11% versus 81.58%; *P* = 0.175). The specificity of GMS was 81.82%, which was higher than that of PAS (81.82% versus 72.73%; *P* = 0.611). The negative predictive value (NPV) for PAS and GMS were 53.33% and 75.00%, respectively. The positive predictive value (PPV) for PAS and GMS were 91.18% and 94.59%, respectively. This study showed that special staining of LCT in BAL fluid may be a novel method for the diagnosis of IPA, and the GMS of LCT had higher sensitivity and specificity, which was superior to PAS.

## 1. Introduction

Invasive pulmonary aspergillosis (IPA) remains a life-threatening disease caused by aspergillus. IPA mainly occurs in immunocompromised patients, including those who have undergone solid organ and stem cell transplantations, those with human immunodeficiency virus (HIV) infection, or those who are receiving long-term immunosuppressive therapy [1–5]. Moreover, IPA has increasingly been found in patients with nonneutropenia, such as patients with chronic obstructive pulmonary disease (COPD) [6], bronchiectasis, and a history of tuberculosis. Air-borne infections caused by *Pneumocystis jirovecii* and *Aspergillus spp.* are frequent. Microbiological diagnosis of *P. jirovecii* infection is commonly based on microscopic detection of cysts and trophic forms [7], so aspergillus spores should be distinguished from *Pneumocystis jirovecii,* cryptococcus spores, and other fungal spores. Mucormycosis and IPA patients have similar underlying diseases, risk factors, clinical manifestations, and radiological signs [8], and the mycelium morphology of mucormycosis is similar to that of aspergillus, so it is easy to mistake mucormycosis for aspergillus. Owing to the nonspecific clinical manifestations, low sensitivity, and specificity of microbiological tests, diagnosing IPA remains a challenge [1, 2, 4]. There is a definite need for a newer method for diagnosis of IPA.

Culturing strains from clinical samples is the main method to diagnose IPA. The lesions of many IPA patients are often located in the periphery of the lung, and the lesions are small, so it is difficult to obtain samples by lung biopsy and bronchial brushing. Even if peripheral pulmonary lesions are acquired by endobronchial ultrasound transbronchial lung biopsy with guide-sheath (EBUS-GS-TBLB), there are few effective ingredients that can be used for microbiology-related examination. However, bronchoalveolar lavage (BAL) fluid obtained by a relatively noninvasive procedure is used for diagnosis of various forms of respiratory diseases [9], so we can perform lavage on peripheral lesions and obtain enough specimens.

The liquid-based cytopathology test (LCT), which was originally applied to gynecologic cervical smears [10], has been progressively used to screen malignant cells in gynecological cytology and nongynecologic body fluid [11, 12], including cervical carcinoma, lung cancer, parathyroid lesions, thyroid cancer, and mesothelioma [13–16]. Compared to conventional cytopathological tests, the LCT not only assists to make cytological diagnosis but also can better protect the cell morphology, the spores, hyphae of the fungus [4, 17, 18] and reduce some technical problems, including a good three-dimensional less air-drying artifact, less obscured by blood, a lower probability of unqualified specimen, and uniform cell thickness [12, 16, 19]. In the current practice, we used the liquid-based cytopathology test of BAL fluid to detect aspergillus. All slides were stained with special staining, including Grocott's methenamine sliver (GMS) and periodic acid-Schiff (PAS). The retrospective study was designed to assess whether LCT used in BAL fluid could provide an additional diagnostic value for IPA and calculate the sensitivity and specificity of PAS and GMS, respectively.

## 2. Methods

### 2.1. Ethical Approval

The study was conducted in accordance with the Declaration of Helsinki and was approved by the Ethics Committee of the Affiliated Hospital of Medical School of Ningbo University, and each patient in the study provided written informed consent.

### 2.2. Patients

A retrospective study was conducted by reviewing the medical records of patients at the Affiliated Hospital of Medical School of Ningbo University from January 2017 to December 2018. A total of 47 consecutive patients who underwent bronchoscopy with BAL fluid and met the possible diagnostic criteria of IPA of the 2017 diagnosis guideline of the European Society for Clinical Microbiology and Infectious Diseases, the European Confederation of Medical Mycology, and the European Respiratory Society (ESCMID-ECMM-ERS) [20] were included in this study. The exclusion criteria were as follows: inadequate BAL fluid specimen (either due to quantity or quality of the specimen) to perform LCT, direct microbial smear and culture, and patients who had BAL fluid performed for a purpose other than evaluation for possible IPA. If the patients underwent multiple BAL fluid analyses within one month, only the first sample was included in the study. If the BAL fluid was collected one month apart, then it was recorded as another sample.

According to the ESCMID-ECMM-ERS guideline [20], there were three levels of probability of IPA: possible, probable, and proven. The diagnosis of possible IPA was based only on clinical symptoms and risk factors. The diagnosis of probable IPA was based on clinical symptoms, risk factors, and mycological evidence, such as positive *Aspergillus sp.* microscopy or culture from sputum or BAL fluid. Diagnosing proven IPA required the histopathological evidence, such as histopathological or cytopathological examination of lung tissue showing hyphae or lung tissue culture positive for *Aspergillus sp*. Patients who met the IPA diagnostic criteria of probable or proven could be diagnosed as having IPA.

### 2.3. Bronchoscopy and Sample Collecting

Experienced doctors performed all bronchoscopies with bronchoalveolar based on recent chest computed tomography (CT) signs. The area of selection of specimens depended on the lesion site of the chest radiograph. If the lesion was limited in the unilateral lung, the bronchoscope would be wedged into the lesion segment. If the diffuse lesions were distributed in the bilateral lung, the bronchoscope would be wedged into the most serious segment of the left and right lung, respectively. 30 ml sterile saline was injected three or four times and the standard was the recovery amount reaching 30 ml, and then the BAL fluid specimens were mixed and centrifuged at 3000 rpm for 5 min, the supernatant was used for Galactomannan (GM) detection, the precipitate was mixed uniformly. A part of the precipitate was used for tuberculosis acid fast staining smear and direct microbial smear (including direct fluorescent staining smears), and the remaining precipitate was inoculated on the blood plate, chocolate agar plate, and Sabouraud Dextrose agar at 35°C for seven days. Then, the results of the culture were observed.

### 2.4. Liquid-Based Cytopathology Test with GMS and PAS

As mentioned above, BAL fluid specimens were prepared and mixed with a mucolytic agent, and the mixture was incubated for 30 min at room temperature and then vortexed for 20 seconds, followed by centrifugation at 600 rpm for 10 min. The supernatant was discarded, and the precipitation was mixed with 10 ml of Tris buffer solution and centrifuged at 600 rpm for 5 min after homogeneous oscillation. The supernatant was removed, vortexed for 20 seconds, and transferred to a slide processor (the AutoCyte PREP system, BD SurePath, Burlington, NC, USA) for automated slide preparation. Finally, GMS and PAS were performed by professional technicians.

### 2.5. Pathological Examination

The samples were analyzed by two cytopathologists who had some experience in microbial morphology and communicated with microbiologists frequently. The cytopathologists wore powder-free gloves during operation to prevent cornstarch powders from contaminating the specimens. At the same time, the respiratory department, microbiology room, and clinicopathological diagnosis center maintained real-time communication. If magenta *Aspergillus* mycelium or other fungal species of mycelium were seen and the cellular response to the pathogens was observed, for example, *Aspergillus mycelia* was often surrounded by neutrophils, and *Cryptococcus* was often phagocytized by macrophages and multinucleated giant cells, the specimens stained with PAS were considered positive. If brown-black Aspergillus mycelium or other fungal species of mycelium were observed and the above mentioned cellular response to the pathogens was seen, the specimens stained with GMS were considered positive.

### 2.6. Statistical Analysis

The sensitivity, specificity, positive predictive value (PPV), negative predictive value (NPV), false-positive rate, and false-negative rate for GMS and PAS were calculated. The differences in sensitivity and specificity between GMS and PAS in the same patient were tested using the *χ*2 test. The categorical variables were presented as counts (percentages), while the continuous data were presented as the mean ± standard deviations (SD). All data were analyzed using SPSS software for Windows, version 18.0 (SPSS). A two-sided *P* < 0.05 indicated statistically significant differences.

## 3. Results

### 3.1. Patient Characteristics

**Figure 1:**
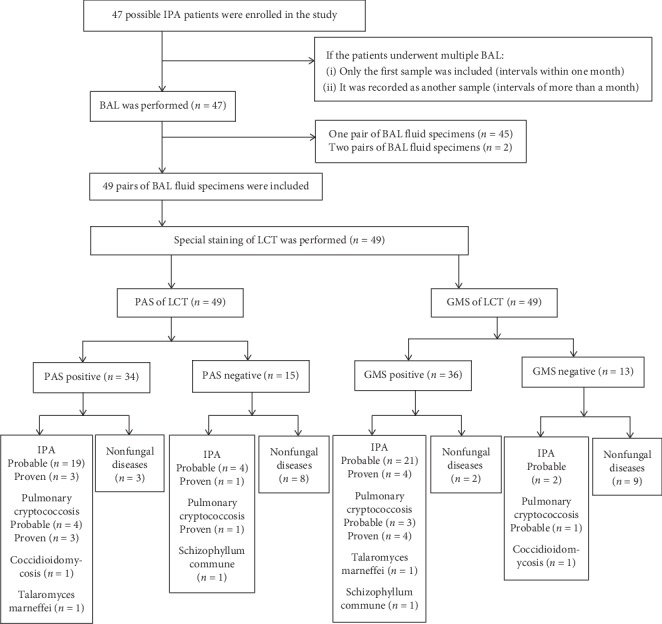
Study flow diagram (2 probable IPA patients had two pairs of BAL fluid specimens). IPA: invasive pulmonary aspergillosis; BAL: bronchoalveolar lavage; LCT: liquid-based cytopathology test; PAS: periodic acid-schiff; GMS: grocott's methenamine sliver.

A total of 47 possible IPA patients were enrolled in our study, duplicate 49 pairs BAL fluid specimens were collected from 47 patients, with 45 people having a pair of BAL fluid specimens and 2 patients having two pairs of BAL fluid specimens (Figure 1). At the same time, 28 biopsy specimens were prepared from the 47 patients underwent bronchoscopic biopsy. According to the diagnostic criteria of ESCMID-ECMM-ERS [20], 25 patients had IPA (21 probable/4 proven), 2 of the 21 probable IPA patients had two pairs of BAL fluid specimens, 11 patients had other invasive fungal diseases, including 8 pulmonary cryptococcosis patients (4 probable/4 proven), 1 *Coccidioidomycosis*, 1 *Schizophyllum commune*, and 1 *Talaromyces marneffei*. Pulmonary cryptococcosis patients met the diagnostic criteria of invasive fungal diseases of the European Organization for Research and Treatment of Cancer and the Mycoses Study Group (EORTC/MSG) [21]. The patient with C*occidioidomycosis* had a short-term travel history in the United States before his illness. After admission, *Coccidioides immitis* were all cultured positive in sputum and BAL fluid, and the mass spectrometer indicated that the patient was positive for *Coccidioides immitis*. *Schizophyllum commune*-induced patient grew flowers and plants at all windows of his home for 20 years before his illness, he had no allergic bronchopulmonary mycosis (ABPM) and no history of other allergic diseases, multiple white colonies were observed after the culture of BAL fluid, and finally, strain identification was confirmed by sequencing the ITS region (ITS-4 of rDNA). Patients with *Talaromyces marneffei* had no history of AIDS,* Talaromyces marneffei* was cultured positive in BAL fluid, and the mass spectrometer indicated that the patient was positive for *Talaromyces marneffei*. 11 patients were diagnosed as having nonfungal diseases, including 3 community acquired pneumonia (CAP), 1 bronchiectasis, 1 lung abscess, 2 tuberculosis and 4 noninfectious disease cases. Attention should be paid to the distinction between invasion and colonization in our study. If the patient had common respiratory symptoms (such as cough, sputum, chest tightness, and shortness of breath) and if the chest radiographic image findings indicated the presence of lesions, the antibacterial treatment was effective, and mycological evidence of the fungus was negative, the patient was considered as having noninvasive fungal disease. In contrast, if the antibacterial treatment was ineffective, microbiological evidence of the fungus was positive, and antifungal therapy was effective, the patient was considered as having invasive fungal diseases.

The characteristics of the 47 patients having underlying conditions and the demographics were shown in Table 1. 13 (27.7%) people had no underlying diseases, among the 34 patients with underlying diseases, 5 (10.6%) had chronic obstructive pulmonary disease, 12 (25.6%) had bronchiectasis, 3 (6.4%) had tuberculosis, 6 (12.8%) had bronchial asthma, 2 (4.3%) had lung cancer, 3 (6.4%) had other solid malignancies, 9 (19.1%) had diabetes, 11 (23.4%) had hypertension, and 3 (6.4%) had hypohepatia.

### 3.2. Cytomorphological Characteristics of Fungal Hyphae

**Figure 2:**
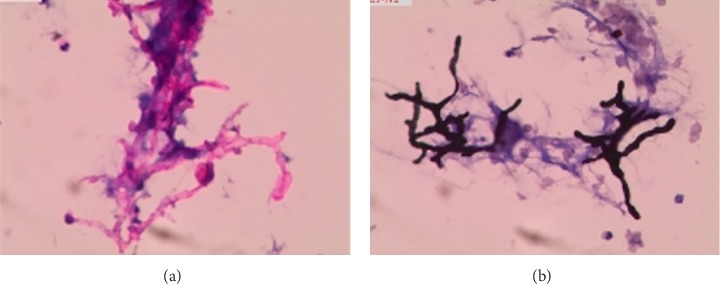
Morphological characteristics of *Aspergillus* in the BAL fluid sample prepared by LCT, followed by special staining. Typical cytological characteristics of aspergillus filament include filament separation, uniform in size, and parallel in wall, with dichotomous branching at acute angles. Magnification, 40x. (a) Magenta filament of LCT in the BAL fluid sample by PAS. (b) Brown-black filament of LCT in the BAL fluid sample by GMS. BAL: bronchoalveolar lavage; LCT: liquid-based cytopathology; PAS: periodic acid-schiff; GMS: grocott's methenamine sliver.

**Figure 3:**
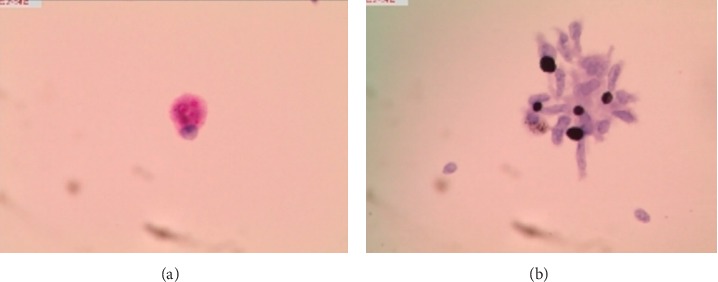
Morphological characteristics of *cryptococcus* in the bronchoalveolar lavage fluid sample prepared by LCT, followed by special staining. *Cryptococcus* was characterized by magenta (PAS) or brown-black (GMS) round, yeast-like organisms. (a) LCT in the bronchoalveolar lavage fluid sample by PAS; (b) LCT in the bronchoalveolar lavage fluid sample by GMS; magnification, 40x. LCT: liquid-based cytopathology; PAS: periodic acid-schiff; GMS: grocott's methenamine sliver.

**Figure 4:**
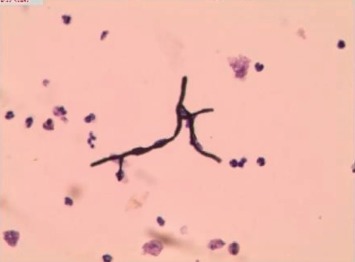
Morphological characteristics of *Schizophyllum commune* in the bronchoalveolar lavage fluid sample prepared by LCT. The mycelium of *schizophyllum* appeared brown-black branches after GMS. Magnification, 40x. LCT: liquid-based cytopathology; GMS: grocott's methenamine sliver.

**Figure 5:**
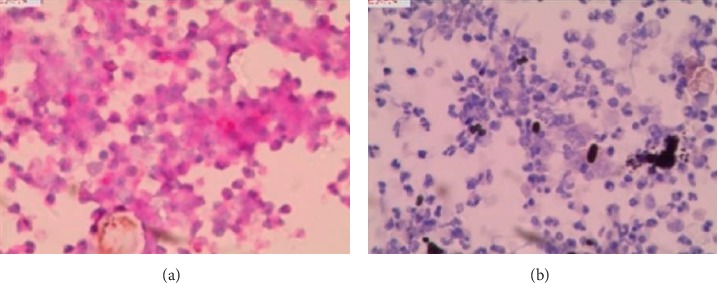
Morphological characteristics of *Talaromyces marneffei* in the bronchoalveolar lavage fluid sample prepared by LCT. Cytological characteristics of *Talaromyces marneffei* include magenta (PAS) or brown-black (GMS) salami spores after special staining. Magnification, 40x. (a) LCT in the bronchoalveolar lavage fluid sample by PAS; (b) LCT in the bronchoalveolar lavage fluid sample by GMS. LCT: liquid-based cytopathology; PAS: periodic acid-schiff; GMS: grocott's methenamine sliver.

**Figure 6:**
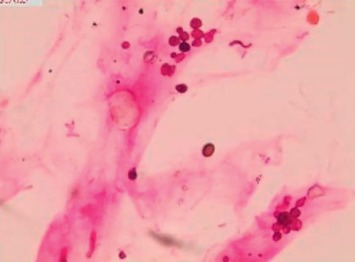
Morphological characteristics of *Coccidioides immitis* in the bronchoalveolar lavage fluid sample prepared by LCT. *Coccidioides immitis* was characterized as a magenta spherical organism after PAS. Magnification, 40x. LCT: liquid-based cytopathology; PAS: periodic acid-schiff.

The slides prepared by LCT could overcome many shortcomings of the conventional smear and made the morphological structure of mycelia and spores clearer. In the BAL fluid specimens prepared by LCT, the mycelia of *Aspergillus* appeared after PAS magenta, filamentous, uniform in size, parallel in wall, with dichotomous branching at acute angles (Figure 2(a)). At the same time, the mycelia of *Aspergillus* presented brown-black after GMS (Figure 2(b)). In the *Aspergillus* positive microbial smear, *Aspergillus* mycelia were observed to be surrounded by neutrophils. In our study, we encountered *Cryptococcus*, *Schizophyllum*, *Talaromyces marneffei*, and *Coccidioides immitis*. C*ryptococcus* with PAS appeared to be round with a diameter of 5∼15 microns, extracellular, magenta, and yeast-like organisms (Figure 3(a)). Similarly, *Cryptococcus* with GMS appeared as a brown-black round capsule (Figure 3(b)). In the *Cryptococcus* positive microbial smear, *Cryptococcus* was observed to be phagocytized by macrophages and multinucleated giant cells. The hyphae of *Schizophyllum* presented as brown-black and branches at an acute angle after being stained with GMS (Figure 4). Unfortunately, the BAL specimens after being stained with PSA were negative in our study. The cell walls of *Talaromyces marneffei* were stained magenta by PAS (Figure 5(a)) and were stained brown-black by GMS (Figure 5(b)), in which a few oblong or elongated sporangia were seen, with blunt circles at both ends. After PAS staining, *Coccidioides immitis* presented magenta spherical organisms which looked like endospores (Figure 6). Unfortunately, after being stained with GMS, it was negative in our study. It should be noted that LCT could find fungal hyphae or spores in BAL fluid but could not distinguish the species of mycelia. The type of hyphae was determined by specimen culture and mass spectrometry.

### 3.3. Performance of LCT Using Special Staining in BAL Fluid

Depending on the diagnostic criteria of ESCMID-ECMM-ERS [20], 25 patients had IPA, and 11 patients had other invasive fungal diseases meanwhile, 11 patients were diagnosed as having nonfungal diseases. Among the 38 BAL fluid samples from 36 patients with invasive fungal diseases, the LCT specimens stained by PAS was positive in 31 (81.58%), and the LCT samples stained by GMS was positive in 35 (92.11%). The sensitivity of GMS was 92.11%, which was higher than that of PAS (92.11% versus 81.58%; *P* = 0.175), but the difference between GMS and PAS was not statistically significant (Table 2). In 27 BAL fluid samples from 25 IPA patients, the positive rate of GMS was higher than that of PAS (92.59% (25/27) versus 81.48% (22/27)) (Table 3). In 8 BAL fluid samples from 8 pulmonary cryptococcosis patients, the positive rate of GMS was the same as that of PAS (87.50%) (Table 3). Among the 11 BAL fluid samples from patients with nonfungal disease, PAS-stained LCT specimens were negative in 8 (72.73%) cases, and GMS-stained LCT specimens were negative in 9 (81.82%) cases. The specificity of GMS was 81.82%, which was higher than that of PAS (81.82% versus 72.73%; *P* = 0.611). The negative predictive value (NPV) for PAS and GMS were 53.33% and 75.00%, respectively. Compared with the NPV of GMS, the NPV of PAS (75.00% versus 53.33%; *P* = 0.247) was lower. The positive predictive value (PPV) for PAS and GMS were 91.18% and 94.59%, respectively. The PPV for GMS was higher than that for PAS (94.59% versus 91.18%; *P* = 0.574) (Table 2). Similarly, there were no significant differences between these groups.

Among 27 BAL fluid samples from 25 IPA patients, the culture of BAL fluid was positive in 18 cases (66.67%) (Table 4), and the direct microbial smear of BAL fluid was positive in 10 cases (37.04%). 14 lung biopsy specimens were prepared from 25 IPA patients, and 3 (21.43%) lung biopsy specimens were positive. In 8 BAL fluid samples from pulmonary cryptococcosis patients, 5 (62.50%) cases were positive in BAL fluid culture, 1 (12.50%) case was positive in the direct microbial smear of BAL fluid, and 4 (80%) cases were positive in 5 lung biopsy specimens from 8 pulmonary cryptococcosis patients (Table 4).

## 4. Discussion

Currently, the morbidity and mortality rates of IPA are gradually increasing, which are related to the delay in early diagnosis of IPA caused by the lack of typical clinical manifestations [22]. Culture and microscopic examination remain the “gold standard” but requires potentially invasive procedures to obtain infected tissue and are insensitive [23–25]. In addition, as mentioned above, attention should be paid to the differentiation of colonization and invasion during IPA diagnosis. In view of the current situation, it is driving the emergence of a novel method to help the diagnosis of IPA.

In recent years, LCT of BAL fluid is commonly used to diagnose lung cancer [26, 27], but it is not commonly done for the detection for aspergillus because the spores and mycelia are easily contaminated by impurities, making it difficult to differentiate mycelia and pseudomycelia, and there is a general lack of knowledge for the cytopathologists on the morphology of fungal infections. The abovementioned factors make LCT of BAL less common for the diagnosis of IPA in clinical practice, but this does not affect LCT of BAL fluid as an auxiliary method for the diagnosis of IPA. In our study, the results showed that the sensitivity and the specificity of GMS in LCT with BAL were all higher than that of PAS. Similarly, the PPV and the NPV of GMS were all superior to that of PAS. On the whole, the special staining of LCT has high sensitivity and specificity, especially GMS, because the mycelia presented by GMS staining are brown-black, which is easier to be observed under the microscope.

In the past studies, the value of LCT in the diagnosis of IPA was rarely studied, especially the LCT of BAL fluid. Shen et al. studied the role of LCT in the diagnosis of IPA in bronchial brushing specimens and sputum specimens and showed that special staining of LCT-prepared slides had a higher positive rate of aspergillus identification for bronchial brushing samples (83.33%) (Table 5) [4]. Similarly, the study by Jiang et al. showed that LCT-prepared slides also had a higher positive rate of *Mucorales* detection for special staining with GMS and PAS in bronchial brushing samples (87.88%) (Table 5) [17]. Meanwhile, Schelenz et al. suggested that special staining methods, such as PAS and GMS, should be used in all available samples to increase the detection rate of fungal hyphae [28]. These studies indicated that special staining of LCT-prepared slides played an important role in the diagnosis of IPA, which was consistent with our experimental results. The difference was that most of the previous studies were about the value of LCT for special staining in bronchial brushing samples or sputum samples, but this time, we studied LCT for special staining in BAL fluid, and it was concluded that LCT specimens with special staining in BAL fluid had higher sensitivity in the diagnosis of IPA, especially GMS.

As mentioned above, special staining of LCT-prepared slides in BAL fluid have many advantages in the process of IPA diagnosis. The slices prepared by LCT and staining are automatically prepared, thus avoiding the interference of practitioner factors. Liquid-based cytology of bronchial aspiration and bronchial brushing materials can even be prepared into cell blocks for the diagnosis of more diseases and molecular tests [29]. However, the limitations of LCT have to be discussed and documented in the literature. First, BAL fluid is vulnerable to contamination, resulting in false positives for specific staining of LCT in BAL fluid, for example, goblet cell mucins are strongly PAS or GMS positive. When the goblet cells are mechanically destroyed, round mucin granules resemble yeast-type fungi. If the clinicians or cytotechnologists wear powdered gloves, cornstarch powders may contaminate the cytology specimens. The distinction from PAS or GMS positive fungal pathogens may be troublesome. Second, although the preparation of LCT slices is not affected by the experience of the participants, microscopic observation of slices must be performed by experienced microbiologists who are familiar with the morphological and structural characteristics of various fungal hyphae. Otherwise, the positive rate of LCT detection will be reduced. Third, mycelia and spores in the slices of LCT in BAL fluid are often observed under a 40-fold microscope, but most pathologists may not observe them carefully under a 40-fold microscope because they are more concerned with cytological benign and malignant morphology than microorganisms. Fourth, the LCT detection of BAL fluid cannot accurately distinguish the type of fungi species, which makes it difficult to choose antifungal drugs. Fifth, there are few studies on the role of LCT for BAL fluid in the diagnosis of IPA at present, and it has not been included in the diagnosis criteria for proven/probable IPA in the international guidelines. Sixth, a major limitation to our study is the small number of patients who had proven/probable IPA, and this may be the reason for the lack of statistical significance between GMS and PAS. Therefore, we should increase the sample size in order to get more accurate experimental data and to further clarify the diagnostic significance of LCT for BAL fluid in IPA.

In our research, our pathologists have some experience in microbiology detection after long-term training and communicate with the respiratory department and microbiology room in time. However, pathologists in other places have not mastered the knowledge of microbiology morphology, so we hope that this technique will be further promoted, and pathologists will communicate with the respiratory department and the microbiology room, which will improve pathologists' ability to diagnose microorganisms under the microscope.

## 5. Conclusion

As a whole, our study shows that LCT-based special staining in BAL fluid seems to be a novel method for the diagnosis of IPA, and the GMS of LCT is superior to PAS, which has higher sensitivity and specificity.

## Figures and Tables

**Table 1:** Clinical characteristics of 47 patients included in the study.

Characteristics	Number of patients
Sex	
Male	33
Female	14
Median age	58.39 ± 11.91
Underlying pulmonary disease	
COPD	5 (10.6%)
Bronchiectasis	12 (25.5%)
Tuberculosis	3 (6.4%)
Bronchial asthma	6 (12.8%)
Lung cancer	2 (4.3%)
Extrapulmonary disease	
Other solid tumor	3 (6.4%)
Diabetes	9 (19.1%)
Hypertension	11 (23.4%)
Hypohepatia	3 (6.4%)
HIV-positive	0
Solid organ transplantation	1 (2.1%)
Corticosteroids treatment	1 (2.1%)
No underlying pulmonary disease	13 (27.7%)

COPD: chronic obstructive pulmonary disease; HIV: human immunodeficiency virus.

**Table 2:** Comparison of the diagnostic efficiency of IPA between the PAS and GMS of LCT in BAL fluid^*∗*^.

	PAS	GMS	*P* value
Sensitivity (%)	81.58	92.11	0.175
Specificity (%)	72.73	81.82	0.611
NPV (%)	53.33	75.00	0.247
PPV (%)	91.18	94.59	0.574
False-positive rate (%)	27.27	18.18	—
False-negative rate (%)	18.42	7.89	—
Positive likelihood ratio	2.99	5.07	—
Negative likelihood ratio	0.25	0.10	—

^*∗*^Sensitivity, specificity, PPV, and NPV were used to compare the diagnostic value of the PAS and GMS of LCT in BAL fluid. IPA: invasive pulmonary aspergillosis; PAS: periodic acid-Schiff; GMS: Grocott's methenamine sliver; LCT: liquid-based cytopathology test; BAL: bronchoalveolar lavage; NPV: negative predictive values; PPV: positive predictive value.

**Table 3:** Results of special staining of liquid-based cytopathology for diagnosing IPA and other invasive fungal diseases.

Invasive fungal diseases	Number of specimens	Special staining	
		GMS	PAS
IPA	27	25 (92.59%)	22 (81.48%)
Pulmonary cryptococcosis	8	7 (87.50%)	7 (87.50%)
Coccidioidomycosis	1	0	1 (100.00%)
Talaromyces marneffei	1	1 (100.00%)	1 (100.00%)
Schizophyllum commune	1	1 (100.00%)	0

IPA: invasive pulmonary aspergillosis; PAS: periodic acid-Schiff; GMS: Grocott's methenamine sliver.

**Table 4:** The positive results of BAL fluid culture, BAL fluid smear, and lung biopsy specimens in diagnosing IPA and other invasive fungal diseases.

Invasive fungal diseases	Number of patients (probable/proven)	Number of specimens	BAL fluid culture positive	BAL fluid smear positive	Lung biopsy specimens	
					Total	Positive
IPA	25 (21/4)	27	18 (66.67%)	10 (37.04%)	14	3 (21.43%)
Pulmonary cryptococcosis	8 (4/4)	8	5 (62.50%)	1 (12.50%)	5	4 (80%)
Coccidioidomycosis	1 (1/0)	1	1 (100%)	0	0	0
Talaromyces marneffei	1 (1/0)	1	1 (100%)	0	0	0
Schizophyllum commune	1 (1/0)	1	1 (100%)	0	1	0

BAL: bronchoalveolar lavage; IPA: invasive pulmonary aspergillosis.

**Table 5:** Comparison of recent studies on the positive rate of LCT in BAL fluid in the diagnosis of IPA.

	Sample type	*n*	Special staining of LCT		
			PAS and GMS both positive	Only PAS positive	Only GMS positive
Shen et al. [4]^a^	Bronchial brushing	54	83.33% (45/54)	—	—
	Sputum	117	43.59% (51/117)	—	—
Jiang et al. [17]^b^	Bronchial brushing	33	87.88% (29/33)	—	—
Our study	BAL fluid	49	—	81.58%	92.11%

^a^Shen et al. studied the role of LCT in the diagnosis of IPA in bronchial brushing specimens and sputum specimens. ^b^Jiang et al. studied the role of LCT in the diagnosis of pulmonary mucormycosis in bronchial brushing samples. LCT: liquid-based cytopathology test; PAS: periodic acid-Schiff; GMS: Grocott's methenamine sliver; BAL: bronchoalveolar lavage; IPA: invasive pulmonary aspergillosis.

## Data Availability

The data used to support the findings of this study are included within the article.
