# Corrigendum to “Special Staining of the Liquid-Based Cytopathology Test in Bronchoalveolar Lavage Fluid for Diagnosis of Invasive Pulmonary Aspergillosis with Nonneutropenic Patients”

**DOI:** 10.1155/2020/7893513

**Published:** 2020-11-06

**Authors:** Yue Hu, Lin Zheng, Deng Pan, Lei Shao, Xianfa Xu, Yiming Yu, Qidong Zhuang, Zhongbo Chen, Zaichun Deng

**Affiliations:** ^1^Department of Pulmonary and Critical Care Medicine, The Affiliated Hospital of Medical School of Ningbo University, 247 Renmin Road, Ningbo, Zhejiang 315020, China; ^2^Department of Microbiology, The Affiliated Hospital of Medical School of Ningbo University, 247 Renmin Road, Ningbo, Zhejiang 315020, China; ^3^Department of Cell, Clinicopathological Diagnosis Center of Ningbo, 685 Huancheng North Road, Ningbo, Zhejiang 315211, China

In the article titled “Special Staining of the Liquid-Based Cytopathology Test in Bronchoalveolar Lavage Fluid for Diagnosis of Invasive Pulmonary Aspergillosis with Nonneutropenic Patients” [1], the author order in the author list was incorrect, where

  Yue Hu, Lin Zheng, Deng Pan, Lei Shao, Xianfa Xu, Yiming Yu, Qidong Zhuang, Zaichun Deng, and Zhongbo Chen should have read:
  Yue Hu, Lin Zheng, Deng Pan, Lei Shao, Xianfa Xu, Yiming Yu, Qidong Zhuang, Zhongbo Chen, and Zaichun Deng

The correct author order is also shown above in the author information.
